# Characterizing Dermatological Conditions in the Transgender Population: A Cross-Sectional Study

**DOI:** 10.1089/trgh.2021.0105

**Published:** 2023-02-08

**Authors:** Suthinee Rutnin, Poonkiat Suchonwanit, Chaninan Kositkuljorn, Cherrin Pomsoong, Sira Korpaisarn, Jiraporn Arunakul, Teerapong Rattananukrom

**Affiliations:** ^1^ Divisions of Dermatology and Department of Internal Medicine, Faculty of Medicine, Ramathibodi Hospital, Mahidol University, Bangkok, Thailand.; ^2^ Divisions of Endocrinology, Department of Internal Medicine, Faculty of Medicine, Ramathibodi Hospital, Mahidol University, Bangkok, Thailand.; ^3^ Division of Child and Adolescent Health, Department of Pediatrics, Faculty of Medicine, Ramathibodi Hospital, Mahidol University, Bangkok, Thailand.

**Keywords:** acne, gender-affirming hormone therapy, hair loss, hirsutism, transgender men, transgender women

## Abstract

**Purpose:**

This study aimed to demonstrate the effects of gender-affirming hormone therapy (GAHT) and gender-affirming procedures on the skin in transgender individuals.

**Methods:**

We conducted a cross-sectional study among transgender people. Skin conditions related to GAHT were assessed, including acne (using the Investigator's Global Assessment, IGA), postacne sequelae, melasma, hypertrichosis in androgen-sensitive areas (HAAs) in transgender men (TM) and hirsutism in transgender women (TW) (using the modified Ferriman–Gallwey score, mFG score), and hair loss (using the Hamilton–Norwood and Ludwig scale) at baseline, 6 months after GAHT, and the day on which the questionnaire was completed. Dermatological problems after gender-affirming procedures were evaluated.

**Results:**

A total of 159 patients, including 134 TM and 25 TW, were eligible to participate. The median duration of GAHT was 23 and 36 months in TM and TW, respectively. In TM, the median IGA score of facial acne increased from 1 at baseline to 3 after 6 months and decreased to 2 after 2 years of GAHT. The mFG score indicated HAA in all TMs after testosterone treatment. A total of 88.1% of TM had no hair loss before hormone therapy. However, after 2 years of GAHT, 76.1% of TM developed male pattern hair loss (MPHL), and 26.1% of them had moderate-to-severe MPHL. In TW, the median IGA and mFG scores decreased after 3 years of hormone therapy, and the proportion of female pattern hair loss (FPHL) in TW increased to 16% after GAHT. In both groups, the most common skin complication after gender-affirming surgery was hypertrophic scarring.

**Conclusions:**

GAHT in TM resulted in acne and MPHL, whereas GAHT in TW caused melasma and FPHL.

## Introduction

The transgender population includes individuals whose gender identity and/or expression differ(s) from their assigned sex at birth, which includes transgender men (TM), transgender women (TW), and transmasculine and transfeminine people. Other people may identify as nonbinary, describing a gender identity and/or expression that fall(s) outside the typical binary categories of male and female.^1,2^ The term TM in this article refers to those whose sex assigned at birth was female and identified as male, and TW refers to those whose sex assigned at birth was male and who identify as female.

The proportion of transgender people in the United States increased by double compared to the previous estimate, from 0.3% in 2011 to 0.6% in 2016.^3,4^ In Asia, the proportion of transgender people is 0.3% in adults.^5^ Nevertheless, there is an underestimated number of transgender people due to variations in terminology, culture, language, and geographic area or a lack of self-reporting and legal protection for the transgender population.^6,7^

Transgender people tend to pursue procedures for physical transformation, including gender-affirming hormone therapy (GAHT) and gender-affirming surgery (GAS) and related procedures. In terms of GAHT, TM receive masculinizing doses of testosterone to induce virilization, whereas feminizing hormones, including estrogen and antiandrogen, are given to TW.^8,9^ Regarding dermatological aspects, androgen and estrogen are known to affect the skin pilosebaceous unit, and both androgen and estrogen receptors are expressed in the sebocytes and hair follicle dermal papilla.^10,11^ Testosterone is converted to dihydrotestosterone (DHT) by the 5α-reductase enzyme in the pilosebaceous unit.^12^ It causes an increase in sebum production and follicular hyperkeratosis, which leads to significant acne vulgaris.^4^ Furthermore, DHT represents the primary regulator of the human hair cycle and exhibits paradoxical effects on hair growth, depending on the gene expression at a particular anatomical site.^11,13^ Although DHT stimulates hair growth on the face and body, it results in hair loss on the scalp.^13,14^ In contrast, estrogen and antiandrogen decrease sebum production, reflecting a relative improvement in acne. The ultimate impact of estrogen on hair growth remains unclear. While estrogen appears to lengthen the anagen phase in pregnant and premenopausal women, *ex vivo* murine models have demonstrated that estrogen agonists promote telogen.^15–18^ Estrogen also stimulates melanogenesis, resulting in melasma.^9,19^ In addition, GAS, including top and bottom surgery, can lead to postoperative scars.^20,21^ Facial affirming procedures, such as neurotoxin and filler injection, may lead to postprocedural complications, particularly those performed by nonmedical personnel.^22–26^

Given the uniqueness and complexities of the transitioning process, transgender individuals may face specific dermatological conditions in addition to routine skincare. Therefore, it is necessary for clinicians who encounter transgender people to recognize these characteristic skin conditions. However, there are limited studies on dermatology issues in transgender people. Most of the available articles were reviews, case reports, and expert opinions.^16,19,27–32^ Published studies regarding dermatological conditions in the transgender population are scarce.^15,33–40^ Thus, this study aimed to demonstrate the proportion of GAHT effects and GAS and facial affirming procedures on the skin in the transgender population.

## Methods

Following approval by the Mahidol University Review Board for Ethics in Human Research (MURA2020/1642), we conducted a questionnaire-based, cross-sectional study in the gender variation clinic at Ramathibodi Hospital, Mahidol University, Thailand from October 2020 to April 2021. The study protocol was performed under the Principles of the Declaration of Helsinki. All included subjects were transgender people who were initiating the first GAHT in the gender variation clinic, were aged ≥18 years old, and were diagnosed with gender dysphoria according to the current Diagnostic and Statistical Manual of Mental Disorders, Fifth Edition. Informed consent was obtained before participation. Individuals with incomplete data and unsigned consent forms were excluded.

Standard digital photographs of the face (front, left, and right side) and hairs (frontal hairline, vertex, and occiput) were taken of all participants. The questionnaire consisted of two main sections. The first section was designed to collect demographic data (age, birth sex, body mass index, income, smoking history, alcohol consumption, and hormone therapy). The treatment route, dosage, treatment duration, and interval of GAHT used were recorded. Adverse effects were documented as increased low-density lipoprotein (LDL), elevated triglycerides, erythrocytosis, and elevated transaminases. The second section focused on dermatological conditions related to GAHT (acne, melasma, hirsutism, and androgenetic alopecia), which were assessed at baseline (before hormone therapy), after 6 months of treatment, and on the day on which the questionnaire was completed.

The Investigator's Global Assessment (IGA) scale for acne and the presence of melasma, as well as the Hamilton–Norwood (H-N) and Ludwig scales for male pattern hair loss (MPHL) and female pattern hair loss (FPHL), respectively, were administered by a treating dermatologist, whereas the modified Ferriman–Gallwey score (mFG score) for hypertrichosis in androgen-sensitive areas (HAAs) in TM or hirsutism in TW was calculated by an endocrinologist at each visit to the gender variation clinic. In addition, skin complications after GAS and facial affirming procedures were recorded.

### Acne and postacne sequelae

Clinical evaluation of acne severity was assessed by a well-accepted IGA scale on the face, different facial areas (forehead, nose, chin, and cheek), and back. The IGA in each location was scored from 0 to 4 (0=clear, 1=almost clear, rare noninflammatory lesions with more than one small inflammatory lesion, 2=mild, some noninflammatory lesions with no more than a few inflammatory papules/pustules (no nodule), 3=moderate, up to many noninflammatory and some inflammatory lesions but no more than one small nodule, and 4=severe, up to many noninflammatory and inflammatory lesions but no more than a few nodules).^41,42^ The proportion of acne was determined by the number of patients who had an IGA score of at least grade 2 at the time of assessment. Postacne sequelae were evaluated by a dermatologist as postinflammatory erythema (PIE), postinflammatory hyperpigmentation (PIH), ice pick, boxcar, rolling, hypertrophic, and keloid scars.^43,44^ Sebum production was self-assessed by the onset of oily skin after facial wash.

### Melasma

We also estimated the proportion of facial melasma in TW. The clinical diagnosis of melasma is light to dark brown hyperpigmented patches with irregular borders on the centrofacial, malar, and mandibular regions.^45^

### Facial and body hair assessment

The mFG score was used for HAA or hirsutism evaluation in TM and TW, respectively. Hirsutism is a term used to describe excessive terminal hairs in an androgen-dependent distribution in women that is mostly associated with hyperandrogenemia.^46^ HAA in males is considered normal and demonstrates male secondary sexual characteristics.

This method is generally used for visually scoring the assessment of facial and body hair growth.^47–49^ The score was determined by hair growth rated from 0 to 4 (0=complete lack of terminal hairs, 1=minimal presence of terminal hairs, 2=more than minimal terminal hairs, 3=not too large hairs, and 4=presence of terminal hairs) in nine different body parts (lip, chin, chest, upper back, sacroiliac region, upper abdomen, lower abdomen, arm, and medial thigh). Total scores <8 are considered normal, and scores ≥8 indicate hirsutism.^47^

### Hair and scalp assessment

The H-N scale and the Ludwig classification were used to evaluate the pattern of hair loss in both TM and TW. Both scales are well-established valid measures of hair loss.^50–53^ The H-N scale assessed MPHL, which was classified from stage I to VII (I: minimal or no recession of the hairline, II: triangular, usually symmetrical areas of recession at the frontotemporal hairline, III: deep symmetrical recession at the temples that are bare or only sparsely covered by hair or primarily hair loss from the vertex with limited recession of the frontotemporal hairline [Type III vertex], IV: more severe frontotemporal recession than in Type III and sparse hair or no hair on the vertex, V: the vertex hair loss region is still separated from the frontotemporal region but it is less distinct, VI: the frontotemporal and vertex regions are joined together, and the extent of hair loss is greater, VII: only a narrow band of hair in a horseshoe shape remains on the sides and back of the scalp). We further subcategorized the H-N scale scores as normal (I), mild (II), and moderate-to-severe MPHL (III-VII).^50–52^ The Ludwig classification for FPHL was divided into type I (minimal thinning), type II (decreased volume and noticeable widening of the midline part), and type III (diffuse thinning with see-through appearance on the top of the scalp).^53^

### Dermatological complications after GAS and facial affirming procedures

The percentage of dermatological complications after GAS and facial affirming procedures (i.e., scarring, infection, wound dehiscence, PIE, and PIH) were evaluated. GAS included top and bottom surgery. The former involved breast mastectomy in TM and breast augmentation in TW, whereas the latter involved genital reassignment surgery. Facial affirming procedures, including botulinum toxin injection, filler injection, and facial GAS (such as rhinoplasty, blepharoplasty, and mid-face osteotomy), were observed.

### Statistical analysis

Normally distributed data are presented as the mean and standard deviation. Non-normally distributed data are shown as the median and interquartile range. Categorical data are presented as numbers and percentages. STATA software version 14.1 (StataCorp, College Station, TX) was used for all analyses. Multivariable logistic regression analyses were performed to evaluate the association between factor variables and dermatological conditions expressed as odds ratios (ORs) with 95% confidence intervals (CIs). Statistical significance was set at *p*-value <0.05.

## Results

A total of 184 individuals were enrolled in the study. Twenty-five patients were excluded due to incomplete questionnaires (*n*=7) and a lack of GAHT (*n*=18). Of the 159 remaining patients, 134 had TM and 25 had TW. Demographic data are summarized in Table 1. The mean age was 31.2±7.2 years in the TM group and 29.3±8.4 years in the TW group. Smoking behavior was considerably more frequently observed in TM than in TW (50% vs. 4%). The median duration of GAHT was 23 and 36 months in TM and TW, respectively.

**Table 1.** Demographic Data Among Transgender Men and Transgender Women

	TM	TW
Number of subjects, *n* (%)	134 (84.3)	25 (15.7)
Age, years±SD	31.2±7.2	29.3±8.4
BMI, kg/m^2^±SD	23.8±4.4	20.9±3.1
History of smoking, *n* (%)	67 (50.0)	1 (4.0)
Smoking, pack/year (IQR)	0.7 (0.1, 1.4)	0.14
Alcohol consumption, *n* (%)	103 (76.9)	8 (32.0)
Income (USD/month), *n* (%)		
<300 USD	13 (9.7)	6 (24.0)
300–600 USD	49 (36.6)	3 (12.0)
600–1200 USD	47 (35.1)	9 (36.0)
>1200 USD	25 (18.7)	7 (28.0)
Serum testosterone levels, ng/dL (IQR)		
Baseline	30 (21, 41)	458 (306, 617)
6 months after GAHT	966 (623.5, 1247.5)	102 (76, 174)
On the survey day	690 (456, 1013)	21 (16, 24)
Serum estradiol levels, pg/mL (IQR)		
Baseline	—	<5
6 months after GAHT	—	72.5 (52, 107)
On the survey day	—	81 (44, 116)
Duration of treatment, months (IQR)	23 (8, 36)	36 (22, 126)
Route of treatment, *n* (%)		
Oral medication	0 (0.0)	14 (56.0)
Topical gel	0 (0.0)	1 (4.0)
Intramuscular medication	134 (100.0)	1 (4.0)
Combined therapy	0 (0.0)	9 (36.0)
Cumulative dose of testosterone, mg (IQR)	4400 (1700, 8000)	—
Cumulative dose of estradiol, mg (IQR)	—	2160 (1140, 10,800)

BMI, body mass index; GAHT, gender-affirming hormone therapy; IQR, interquartile range; SD, standard deviation; TM, transgender men; TW, transgender women; USD, United States dollar.

Regarding testosterone therapy (Table 2), all TM patients received hormonal treatment through the intramuscular route, whereas most TW patients received the oral route. In TM, 96.3% used testosterone enanthate with a mean dosage of 130.8 mg and a mean interval of 2.4 weeks, whereas 3.7% used 1000 mg of testosterone undecanoate with a mean interval of 12 weeks. Increased LDL levels were the most common side effect, followed by erythrocytosis in testosterone enanthate and testosterone undecanoate (50.4% and 60%, 32.6%, and 40%, respectively). Elevated triglyceride levels and transaminases were found only in testosterone enanthate (7% and 3.1%, respectively).

**Table 2.** Hormone Therapy in Transgender Men

	Testosterone enanthate	Testosterone undecanoate
Number of patients, *n* (%)	129 (96.3)	5 (3.7)
Dose, mean±SD	130.8±58.3	1000±0.0
Interval, weeks mean±SD	2.4±0.8	12±1.6
Side effects, *n* (%)		
Increased LDL levels (>130 mg/dL)	65 (50.4)	3 (60.0)
Elevated TG levels (>150 mg/dL)	9 (7.0)	0 (0.0)
Erythrocytosis (Hct >50%)	42 (32.6)	2 (40.0)
Elevated transaminases (>3 folds UNL)	4 (3.1)	0 (0.0)

Hct, hematocrit; LDL, low-density lipoprotein; TG, triglycerides; UNL, upper normal limit.

Regarding estrogen and antiandrogen therapy in TW (Table 3), 92% used oral estradiol valerate, and 64% used an oral combination of estradiol valerate and cyproterone acetate (Supplementary Table S1). Topical estradiol gel and gonadotropin-releasing hormone agonists were used in 24% and 4% of patients, respectively. Only one participant who received estradiol valerate had increased triglyceride levels.

**Table 3.** Hormone Therapy in Transgender Women

	n=25
Estrogen (estradiol valerate), *n* (%)	23 (92.0)
Estrogen (estradiol valerate) and antiandrogen (cyproterone acetate), *n* (%)	16 (64.0)
GnRH agonist, *n* (%)	1 (4.0)
Estradiol gel, *n* (%)	6 (24.0)
Side effects, *n* (%)	
Elevated TG levels (>150 mg/dL)	1 (4.0)
Venous thromboembolism	0 (0.0)
Osteoporosis	0 (0.0)

GnRH, gonadotropin releasing hormone.

### Dermatological conditions in TM

#### Acne and postacne sequelae

The median IGA scores of facial acne were 1 at baseline, 3 after 6 months of GAHT, and 2 on the day of the survey (Table 4). The questionnaire was administered a median of ∼2 years after GAHT. Before GAHT, the proportion of facial acne was 28.4%. It increased to 87.5% and decreased to 60.4% after 6 months and 2 years of GAHT. The IGA scores on the back increased from 0 at baseline to 2 after 6 months and 2 years of GAHT. The proportion of back acne was 15.7% at baseline. It increased to 78.3% and decreased to 54.5% after 6 months and 2 years of GAHT, respectively. The common postacne sequelae were PIE and PIH. The median onset of sebum production was reduced from 3 h at baseline to 2 h after 6 months and 2 years of GAHT.

**Table 4.** Dermatological Conditions in Transgender Men

	Baseline	6 months after GAHT	On the survey day
Number of patients	134	120	134
Acne			
Proportion of acne, *n* (%)	38 (28.4)	107 (89.2)	99 (73.9)
Proportion of facial acne, *n* (%)	38 (28.4)	105 (87.5)	81 (60.4)
Proportion of back acne, *n* (%)	21 (15.7)	94 (78.3)	73 (54.5)
IGA on the face, median (IQR)	1 (1, 2)	3 (2, 3)	2 (1, 2)
Forehead	0 (0, 1)	1 (1, 2)	1 (1, 1)
Nose	1 (0, 1)	1 (1, 2)	0 (0, 1)
Chin	1 (0, 1)	2 (1, 3)	1 (1, 2)
Cheek	0 (0, 1)	2 (1, 3)	1 (1, 2)
IGA on the back, median (IQR)	0 (0, 1)	2 (1, 3)	2 (1, 3)
Treatment of acne, *n* (%)	31/38 (81.6)	66/107 (61.7)	75/99 (75.8)
Topical medication	25/31 (80.6)	50/66 (75.8)	57/75 (76.0)
Oral medication	7/31 (22.6)	14/66 (21.2)	10/75 (13.3)
Intralesional corticosteroid	8/31 (25.8)	15/66 (22.7)	13/75 (17.3)
Laser and energy-based devices	5/31 (16.1)	7/66 (10.6)	11/75 (14.7)
Postacne sequelae, *n* (%)	37/38 (97.4)	80/107 (74.8)	96/99 (97.0)
PIE	24/37 (64.9)	50/80 (62.5)	56/96 (58.3)
PIH	22/37 (59.5)	43/80 (53.8)	59/96 (61.5)
Ice pick scar	11/37 (29.7)	22/80 (27.5)	31/96 (32.3)
Rolling scar	6/37 (16.2)	17/80 (21.3)	17/96 (17.7)
Box scar	5/37 (13.5)	8/80 (10.0)	8/96 (8.3)
Hypertrophic scar	0/37 (0.0)	6/80 (7.5)	3/96 (3.1)
Sebum production, hours (IQR)	3 (2.0, 5.5)	2 (1.0, 3.0)	2 (1.0, 3.0)
Hair			
mFG score, median (IQR)	2 (2.0, 12.0)	15.5 (12.0, 18.5)	16.5 (12.0, 20.0)
Absence of HAA (<8), *n* (%)	82 (61.2)	0 (0.0)	0 (0.0)
Presence of HAA (≥8), *n* (%)	52 (38.8)	120 (100.0)	134 (100.0)
Treatment of HAA, *n* (%)	33/52 (63.5)	35/120 (29.2)	43/134 (32.1)
Shaving	31/33 (93.9)	33/35 (94.3)	41/43 (95.3)
Topical medication	2/33 (6.1)	1/35 (2.9)	1/43 (2.3)
Waxing	1/33 (3.0)	1/35 (2.9)	1/43 (2.3)
Laser and energy-based devices	1/33 (3.0)	0/35 (0.0)	0/43 (0.0)
Hamilton–Norwood classification, *n* (%)			
Normal (I)	118 (88.1)	70 (58.4)	30 (22.4)
Mild MPHL (II)	5 (3.7)	31 (25.8)	67 (50.0)
Moderate to severe MPHL (III–VII)	11 (8.2)	19 (15.8)	35 (26.1)
Mixed type hair loss (MPHL+FPHL)	—	—	2 (1.5)
Treatment of MPHL with topical minoxidil	2/16 (12.5)	5/50 (10.0)	10/104 (9.6)

FPHL, female pattern hair loss; HAA, hypertrichosis in androgen-sensitive area; IGA, Investigator's Global Assessment; mFG score, modified Ferriman–Gallwey score; MPHL, male pattern hair loss; PIE, postinflammatory erythema; PIH, postinflammatory hyperpigmentation.

#### Hair assessment

At baseline, the median mFG score on facial and body hair was 2; it increased to 15.5 and 16.5 after 6 months and 2 years of GAHT, respectively. This indicated an increase in the amount/density of body hair, which is often a desirable effect of testosterone for TM. Regarding scalp hair, the majority of TM (88.1%) had no hair loss before testosterone treatment, whereas 3.7% and 8.2% had mild MPHL and moderate-to-severe MPHL, respectively. After 2 years of GAHT, 76.1% of TM developed MPHL, and 26.1% of TM progressed to moderate-to-severe MPHL. Interestingly, 1.5% TM resulted in mixed MPHL-FPHL-type hair loss.

### Dermatological conditions in TW

#### Acne and postacne sequelae

The median IGA score of facial acne decreased from 2 at baseline to 1 after 6 months of GAHT and on the day of the survey, which was 3 years after GAHT (Table 5). Before hormone therapy, the proportion of facial acne was 64%. It decreased to 31.8% and 8% at 6 months and 3 years of GAHT, respectively. Regarding back acne, the median IGA score was 0 at baseline and remained constant after GAHT. The proportion of back acne at baseline was 16%, which slightly increased to 27.3% after 6 months and diminished to 20% after 3 years of GAHT. Similar to TM, the common postacne sequelae were PIE and PIH. The median onset of sebum production was increased from 1 h at baseline to 3 h after 6 months and 3 years of GAHT.

**Table 5.** Dermatological Conditions in Transgender Women

	Baseline	6 months after GAHT	On the survey day
Number of patients	25	22	25
Acne			
Proportion of acne, *n* (%)	16 (64.0)	7 (31.8)	5 (20.0)
Proportion of facial acne, *n* (%)	16 (64.0)	7 (31.8)	2 (8.0)
Proportion of back acne, *n* (%)	4 (16.0)	6 (27.3)	5 (20.0)
IGA on the face, median (IQR)	2 (1, 3)	1 (0, 2)	1 (0, 1)
Forehead	1 (0, 3)	0 (0, 1)	0 (0, 1)
Nose	1 (0, 2)	0 (0, 1)	0 (0, 0)
Chin	1 (0, 2)	0 (0, 1)	0 (0, 1)
Cheek	0 (0, 1)	0 (0, 0.5)	1 (0, 1)
IGA on the back, median (IQR)	0 (0, 1)	0 (0, 0.5)	0 (0, 0)
Treatment of acne, *n* (%)	13/16 (81.3)	3/7 (42.9)	3/5 (60.0)
Topical medication	13/13 (100.0)	3/3 (100.0)	3/3 (100.0)
Oral medication	2/13 (15.4)	1/3 (33.3)	1/3 (33.3)
Intralesional corticosteroid	3/13 (23.1)	1/3 (33.3)	1/3 (33.3)
Laser and energy-based devices	2/13 (15.4)	1/3 (33.3)	1/3 (33.3)
Postacne sequelae, *n* (%)	14/16 (87.5)	5/7 (71.4)	3/5 (60.0)
PIE	11/14 (78.6)	4/5 (80.0)	2/3 (66.7)
PIH	10/14 (78.4)	3/5 (60.0)	0/3 (0.0)
Ice pick scar	1/14 (7.1)	1/5 (20.0)	2/3 (66.7)
Rolling scar	1/14 (7.1)	2/5 (40.0)	2/3 (66.7)
Box scar	0/14 (0.0)	1/5 (20.0)	2/3 (66.7)
Hypertrophic scar	0/14 (0.0)	0/5 (0.0)	0/3 (0.0)
Sebum production, hours (IQR)	1 (1, 3)	3 (1, 5)	3 (1, 6)
Melasma			
Melasma, *n* (%)	3 (12.0)	7 (31.8)	7 (28.0)
Hair			
mFG score, mean±SD	13.2±4.8	10.0±4.3	10.5±2.6
Absence of hirsutism (<8), *n* (%)	0 (0.0)	0 (0.0)	0 (0.0)
Presence of hirsutism (≥8), *n* (%)	25 (100.0)	22 (100.0)	25 (100.0)
Treatment of hirsutism, *n* (%)	16/25 (64.0)	15/22 (68.2)	15/25 (60.0)
Shaving	10/16 (62.5)	8/15 (53.3)	7/15 (46.7)
Topical medication	1/16 (6.3)	2/15 (13.3)	2/15 (13.3)
Waxing	7/16 (43.8)	5/15 (33.3)	5/15 (33.3)
Laser and energy-based devices	5/16 (31.3)	4/15 (26.7)	6/15 (40.0)
Ludwig classification, *n* (%)			
Ludwig type I	0 (0.0)	1 (4.5)	2 (8.0)
Ludwig type II	0 (0.0)	0 (0.0)	2 (8.0)
Mixed type hair loss (MPHL+FPHL)	—	—	3 (12.0)
Treatment of FPHL with topical minoxidil	0 (0.0)	1/1 (100.0)	3/7 (42.9)

#### Melasma

Twelve percent of TW had melasma at baseline. The number increased to 31.8% at 6 months and steadily decreased to 28% after 3 years of GAHT.

#### Hair assessment

Hirsutism was found in all TW, both before and after GAHT, as all TW had been exposed to testosterone throughout puberty until receiving estrogen and/or antiandrogen therapy in the third decade of life. However, the severity of hirsutism decreased as the mFG score decreased from 13.2 at baseline to 10 after 6 months of GAHT and persisted at 10.5 after 3 years of GAHT. A minority of TW (4.5%) had FPHL 6 months after GAHT. However, after 3 years of GAHT, the proportion of FPHL increased to 16%, in which Ludwig types I and II were equally found in 8% of cases. In addition, FPHL combined with MPHL was observed in 12% of cases.

### Dermatological complications after GAS and facial affirming procedures

TW had facial affirming procedures more than TM, and these procedures included botulinum toxin injection (44% and 9%), filler injection (24% and 3.7%), and facial GAS (28% and 17.9%) (Table 6). However, there were no reports of skin complications after botulinum toxin and filler injection. Only 12.5% of patients reported hypertrophic scars from rhinoplasty.

**Table 6.** Gender Affirming Procedures in Transgender People

	TM (*n*=134)	TW (*n*=25)
Facial affirming procedure		
Botulinum toxin injection, *n* (%)	12 (9.0)	11 (44.0)
Forehead	8/12 (66.7)	4/11 (36.4)
Crow's feet	6/12 (50.0)	5/11 (45.5)
Masseter	5/12 (41.7)	8/11 (72.7)
Facelift	4/12 (33.3)	4/11 (36.4)
Facial GAS, *n* (%)	24 (17.9)	7 (28.0)
Rhinoplasty	22/24 (91.7)	3/7 (42.9)
Blepharoplasty	3/24 (12.5)	3/7 (42.9)
Mid-face osteotomy	0/24 (0.0)	1/7 (14.3)
Filler injection, *n* (%)	5 (3.7)	6 (24.0)
Gender affirming surgery		
Top surgery, *n* (%)	50 (37.3)	9 (36.0)
Bottom surgery, *n* (%)	19 (14.2)	8 (32.0)
Hysterectomy and oophorectomy	19/19 (100.0)	—
Orchidectomy	—	8/8 (100.0)
Vulvovaginoplasty and clitoroplasty	—	6/8 (75.0)

GAS, gender-affirming surgery.

For GAS, 37.3% of TM patients underwent mastectomy, whereas only 14.2% underwent bottom surgery, including hysterectomy and oophorectomy. In contrast, the proportions of breast augmentation and bottom surgery were similar in TW (36% and 32%, respectively). Considering postsurgical complications, hypertrophic scarring (24%), PIH (10%), PIE (8%), atrophic scarring (8%), keloid scarring (4%), and wound dehiscence (2%) were observed in TM. In TW, only 16.7% of patients developed hypertrophic scars, and 8.3% had keloid scars.

### Factors associated with dermatological conditions in TM and TW

To determine the predictive factors of dermatological conditions in TM and TW, logistic regression analysis was performed, and the results are reported in Table 7. In univariate analyses, MPHL in TM was significantly associated with the duration of testosterone therapy (OR: 1.04, 95% CI: 1.01–1.08, *p*=0.005). After adjusting for the relevant confounding factors, the duration of testosterone therapy remained a predictive risk factor for the development of MPHL (OR: 1.05, 95% CI: 1.01–1.09, *p*=0.016). There were no factors significantly associated with other dermatological conditions, including acne, melasma, and FPHL, in either TM or TW. HAA and hirsutism were the two conditions that were represented in all TM and TW at the time of the survey; thus, the association was unable to be assessed.

**Table 7.** Factors Associated with Dermatological Conditions in Transgender Men and Transgender Women

	TM		TW	
	Adjusted OR (CI)^a^	*p*	Adjusted OR (CI)^a^	*p*
Acne				
Age, years	0.97 (0.92–1.03)	0.288	1.04 (0.90–1.22)	0.532
BMI ≥25	0.94 (0.38–2.34)	0.896	—	—
History of smoking	1.21 (0.52–2.83)	0.660	—	
Alcohol consumption	1.46 (0.55–3.83)	0.446	1.04 (0.06–19.22)	0.978
Hormonal duration, months	1.01 (0.98–1.04)	0.369	1.02 (0.98–1.06)	0.366
Cumulative dose of testosterone, mg	1.00 (1.00–1.00)	0.786	—	**—**
Cumulative dose of estradiol, mg	—	—	1.00 (1.00–1.00)	0.366
Melasma				
Age, years	—	—	1.18 (0.96–1.42)	0.126
BMI ≥25 (overweight)	—	—	—	—
History of smoking	—	—	—	
Alcohol consumption	—	—	0.02 (0.00–5.19)	0.171
Hormonal duration, months	—	—	0.98 (0.94–1.02)	0.273
Cumulative dose of testosterone, mg	—	—	—	—
Cumulative dose of estradiol, mg	—	—	1.00 (1.00–1.00)	0.178
Hair loss				
Age, years	1.02 (0.97–1.09)	0.423	1.06 (0.91–1.22)	0.451
BMI ≥25 (overweight)	0.75 (0.28–1.98)	0.563	—	—
History of smoking	1.11 (0.40–2.51)	0.992	—	
Alcohol consumption	0.66 (0.21–2.05)	0.475	0.88 (0.06–13.79)	0.930
Hormonal duration, months	1.05 (1.01–1.09)	0.016^b^	0.99 (0.96–1.03)	0.706
Cumulative dose of testosterone, mg	1.00 (1.00–1.00)	0.906	—	—
Cumulative dose of estradiol, mg	—	—	1.00 (1.00–1.00)	0.542

^a^ Includes age, BMI, history of smoking, alcohol consumption, hormonal duration, and cumulative dose of hormonal therapy.

^b^ *p*-Value <0.05 was considered statistically significant.

CI, confidence interval; OR, odds ratio.

## Discussion

To date, more recent population-based studies have found a 10- to 100-fold increase in the number of transgender people.^3,6,7^ GAHT appears to be the first-line hormone therapy to allow them to express their gender identity; however, such treatment could yield adverse results. This study aimed to summarize the dermatological conditions related to GAHT and gender-affirming procedures and improve care for transgender populations.

In our study, testosterone caused a rapid increase in facial and back acne in TM. The proportion of facial acne increased from 28.4% to 87.5% within 6 months and then decreased to 60.4% after 2 years of GAHT. These results were consistent with previous studies.^15,34^ Wierckx et al. reported an increase in facial acne from 35% at baseline to 82% after 6 months and 55% after 1 year of GAHT (*p*=0.026).^34^ Giltay et al. showed an increased facial acne proportion from 29.4% at baseline to 94% after 4 months of hormone therapy (*p*=0.005).^15^ Similarly, the proportion of back acne in our study increased from 15.7% to 78.3% at 6 months and decreased to 54.5% after 2 years of GAHT. Wierckx et al. demonstrated that back acne increased from 15% at baseline to 88% after 6 months and 50% after 1 year of GAHT (*p*=0.001).^34^ Based on previous studies, acne lesions occurred within the first 6–9 months and decreased at 12 months after hormone therapy.^15,34^ This may be explained by the initial increase in sebum production after testosterone treatment, and testosterone levels attenuate over time.^15^ In our study, 75% of the patients received acne treatment during GAHT, which may have contributed to acne improvement. Regarding mild acne (IGA=2) in the TM after GAHT, most of the patients (75.8%) received only topical medication.

Treatments for acne in TM are similar to standard treatment guidelines in the general population. Topical retinoid and/or benzoyl peroxide are mainstay treatments for mild acne. Systemic antibiotics, especially tetracyclines, are prescribed in addition to topical treatment for moderate and severe acne. In severe, refractory, or nodulocystic acne, the concomitant use of oral isotretinoin is considered.^27,28,54–56^ However, combining testosterone with tetracyclines or isotretinoin may increase the risk of hepatotoxicities and requires closer monitoring of these patients. In addition, an isotretinoin prescription must be carefully considered among TW who do not have GAS, as it has a teratogenic effect. Hormone therapy, including oral contraceptive pills and spironolactone, may counter the desired masculinizing effects of testosterone and is not recommended in these men.^27,28^

With regard to hair growth, testosterone increases the hair growth rate, hair density, and hair diameter of facial and body hair, leading to HAA after GAHT in TM.^15^ Our study demonstrated a rapid change in the mFG score from the absence to the presence of HAA after 6 months of GAHT, which remained steady at ∼2 years. This was in agreement with the results of previous studies showing that the development of HAA occurred after 1 year of GAHT (*p*<0.001).^15,34^ In contrast to facial and body hair, DHT induces dermal papillae apoptosis and stimulates shortening of the anagen phase and hair follicle miniaturization of the scalp region of genetically predisposed people.^14^ In our study, half of the TM patients had mild MPHL, whereas one-fourth of TM patients developed moderate-to-severe MPHL after 2 years of GAHT. Compared to a previous study, mild MPHL and moderate-to-severe subtype were found equally in one-third of TM after long-term hormone therapy.^34^ In general, the duration of MPHL after GAHT ranged from 2 to 5 years.^57^

A mixed pattern of MPHL and FPHL was found in 1.5% of patients in our study. This could be present in TM due to their initial sex hormone derivative and after receiving GAHT, resulting in the specific stereotype pattern of hair loss.^58^ The duration of testosterone therapy was only identified as a significant factor associated with MPHL in TM (OR: 1.05, 95% CI: 1.01–1.09, *p*=0.016). Although MPHL is a well-known multifactorial disease, our study suggests that a longer duration of testosterone exposure may initiate the development of the disease among TM patients, who were considered originally naive to male sex hormones.

Currently, the Food and Drug Administration has approved only three medical treatments for this condition, including topical minoxidil and low-level laser light therapy for both MPHL and FPHL and oral finasteride for MPHL.^58^ Topical 5% minoxidil foam or solution twice daily is recommended for mild MPHL in men. However, no study has investigated the dosing schedule of minoxidil in transgender people. Dustin et al. recommended starting 5% minoxidil twice daily in both TM and TW.^57^ While oral finasteride represents one of the most effective treatments in moderate-to-severe MPHL, the usage of this drug should be recommended with caution, as it may interfere with the development of desired secondary sex characteristics. A prospective cohort study demonstrated that oral finasteride 1 mg daily can be prescribed with clinical improvement for MPHL in TM after at least 2 years of testosterone therapy.^57^

In TW, estrogen and antiandrogen are chosen to be baseline GAHT for blocking androgen production.^59^ Our study demonstrated the improvement in IGA score in TW in terms of facial and back acne after GAHT. The disappearance of acne in all TW patients following 4 months of estrogen administration was reported.^15^ Another cross-sectional study showed an improvement of moderate-to-severe acne in TW from 13.7% to 0.9% after receiving GAHT (*p*<0.001).^35^ However, in TW with acne recalcitrant to hormone therapy, endocrine evaluation for hyperandrogenic states should be considered.^4^

The prevalence of melasma in individuals who were assigned male sex at birth without previous hormonal treatment in our study was 12%, consistent with the 13.6% of melasma in men in the Chinese population^60^ and 14.5% among male Latino migrant workers in the United States.^61^ Nevertheless, it is a lower prevalence compared to the Indian male population (20.5%).^62^ This indicates that melasma in men is more common in individuals with dark skin with Fitzpatrick skin types IV–VI.^61,62^ In addition, a higher prevalence of melasma among Hispanic, Asian, and African Americans was found to be more common than in the White population.^63,64^ There were reports of melasma in TW receiving hormones for a period of 18 months.^19^ In our study, melasma was found in one-third of participants, and the proportion was similar compared to cisgender women (28% vs. 29.9%).^65^

Estrogen can change facial and body hair patterns. Decreasing mFG scores from baseline to 6 months and 3 years after GAHT were observed in our study. Similarly, Giltay et al. reported a progressive decrease in the mFG score from 21 to 10 after 12 months of GAHT (*p*<0.001). Moreover, TW experienced decreasing hair diameter, a terminal hair growth rate, and density within 4 months, which remained constant.^15^

The proportion of FPHL in our study increased from 0% at baseline to 16% at the time of the survey. To the best of our knowledge, this is the first study demonstrating an increase in FPHL proportion among TW. It has been postulated that estrogen stimulates hair growth by prolonging the anagen phase.^58^ In contrast, some experimental studies observed hair loss after estrogen agonist administration.^18,66–68^ However, at present, an increased estrogen-to-androgen ratio might be the best explanation for increased FPHL in TW because hypotestosteronemia was found to be more prevalent in male patients presenting with FPHL, and TW receiving GAHT are considered the estrogen-to-androgen ratio of elevated individuals.^69^ Differences in the estrogen and androgen levels in each scalp area may contribute to a different pattern of hair loss presentations between MPHL and FPHL.^16,70^ Our study noted the presence of a mixed-type pattern of MPHL and FPHL in 12% of TW. MPHL in TW can result from androgen-dependent and genetically susceptible individuals, as described in the common pathogenesis of androgenetic alopecia^53^; once treated with GAHT, the estrogen:androgen ratio is relatively increased, leading to a combined MPHL-FPHL presentation. Thus, physicians should raise awareness when prescribed estrogen therapy because of these findings. Topical minoxidil 2% solution and 5% foam have been approved to treat FPHL. Oral administration of finasteride or dutasteride is contraindicated for cisgender women with FPHL. However, it could be safe for FPHL in TW due to the antiandrogen effect.^27,58,71^

There are some limitations of this study. First, recall bias and subjective assessment of some information occurred. Second, there was no control group of cisgender men and women to compare the baseline proportions of these dermatological conditions. Third, the number of TW participants was relatively small and did not represent the general TW population. Fourth, dermatological conditions related to GAHT in TM in the study were not transferable to other clinical populations because the majority of participants received testosterone enanthate.

## Conclusions

After hormone therapy, TM were more likely to have acne and MPHL, whereas TW commonly developed melasma and FPHL. This is the first study exploring dermatological conditions after GAHT in Southeast Asia and covering almost all aspects of skin findings related to GAHT in transgender people. Due to geographic differences and cultural restrictions, variable information may emerge as a new result. In our study, mixed MPHL-FPHL-type hair loss was a novel dermatological finding in both TM and TW. Further prospective longitudinal studies with a large number of subjects with each dermatological concern and associated variable factors, such as family history and genetic predisposition, are recommended.

## Supplementary Material

Supplemental data

## Abbreviations Used

BMI: body mass index
CI: confidence interval
DHT: dihydrotestosterone
FPHL: female pattern hair loss
GAHT: gender-affirming hormone therapy
GAS: gender-affirming surgery
GnRH: gonadotropin releasing hormone
HAA: hypertrichosis in androgen-sensitive area
Hct: hematocrit
H-N: Hamilton–Norwood
IGA: Investigator's Global Assessment
IQR: interquartile range
LDL: low-density lipoprotein
mFG score: modified Ferriman–Gallwey score
MPHL: male pattern hair loss
OR: odds ratio
PIE: postinflammatory erythema
PIH: postinflammatory hyperpigmentation
SD: standard deviation
TG: triglycerides
TM: transgender men
TW: transgender women
UNL: upper normal limit
USD: United States dollar
