# Supplementary material for: Characterizing Dermatological Conditions in the Transgender Population: A Cross-Sectional Study
Source: Transgend Health. 2023 Feb 8;8(1):89–99. doi: 10.1089/trgh.2021.0105 (PMC9942180; doi:10.1089/trgh.2021.0105)
Supplement: Supplemental data [file Supp_TableS1.docx]

**Supplementary Table S1. Comparison of transgender women receiving estrogen with and without antiandrogen therapy**

| **Factors** | **Estradiol valerate** | **Estradiol valerate with cyproterone acetate** | ***P* value** |
| --- | --- | --- | --- |
| Number of subjects, *n* *(%)* | 9 (36.0) | 16 (64.0) |  |
| Age, years ± SD | 34.3 ± 2.4 | 26.5 ± 2.0 | 0.022^a^ |
| BMI, kg/m^2^ ± SD | 21.9 ± 3.9 | 20.3 ± 2.4 | 0.218 |
| History of smoking, *n (%)* | 1 (11.1) | 0 (0.0) | 0.360 |
| Alcohol consumption, *n (%)* | 5 (55.6) | 3 (18.8) | 0.075 |
| Hormonal duration, months (IQR) | 36 (24, 126) | 36.5 (19, 120) | 0.825 |
| Acne |  |  |  |
| Proportion of facial acne, *n (%)* | 0 (0.0) | 2 (12.5) | 0.400 |
| Proportion of back acne, *n (%)* | 3 (33.3) | 2 (12.5) | 0.230 |
| IGA on the face, median (IQR) | 0 (0, 1) | 1 (0, 1) | 0.233 |
| IGA on the back, median (IQR) | 0 (0, 1) | 0 (0, 0) | 0.239 |
| Melasma |  |  |  |
| Melasma, *n (%)* | 3 (33.3) | 4 (25.0) | 0.499 |
| Hair |  |  |  |
| Modified Ferriman-Gallwey score, mean ± SD | 11.3 ± 3.2 | 10.1 ± 2.1 | 0.246 |
| Presence of hirsutism, *n (%)* | 9 (100.0) | 16 (100.0) | 1 |
| Presence of FPHL, *n (%)* | 1 (12.5) | 6 (37.5) | 0.218 |
| Hormonal levels |  |  |  |
| Low estradiol levels (< 100 pg/mL), *n (%)* | 4 (44.5) | 13 (81.2) | 0.152 |
| Estradiol levels (100 – 200 pg/mL), *n (%)* | 2 (22.2) | 1 (6.3) |  |
| High estradiol levels (> 200 pg/mL), *n (%)* | 3 (33.3) | 2 (12.5) |  |
| Low testosterone levels (< 50 ng/dL), *n (%)* | 8 (88.9) | 15 (93.8) | 0.600 |
| Gender affirming procedures |  |  |  |
| Facial affirming procedure, *n (%)* | 2 (22.2) | 5 (31.3) | 0.501 |
| Gender affirming surgery, *n (%)* | 8 (88.9) | 4 (25.0) | 0.003^a^ |
| Top surgery, *n (%)* | 5 (55.6) | 4 (25.0) | 0.137 |
| Bottom surgery, *n (%)* | 7 (77.8) | 1 (6.3) | 0.001^a^ |
| - Orchiectomy | 7 (77.8) | 1 (6.3) | 0.001 ^a^ |
| - Vulvovaginoplasty and clitoroplasty | 5 (55.6) | 1 (6.7) | 0.015 ^a^ |

^a^ *P* value < 0.05 was considered statistically significant.

BMI, Body mass index; FPHL, Female pattern hair loss; IGA, Investigator Global Assessment; IQR, Interquartile range; kg/m^2^, Kilogram per square meter; mg, Milligram; ng/dL, Nanograms per deciliter; pg/mL, Picograms per milliliter; SD, Standard deviation.
